# Associative Learning Beyond the Medial Temporal Lobe: Many Actors on the Memory Stage

**DOI:** 10.3389/fnbeh.2013.00162

**Published:** 2013-11-19

**Authors:** Giulio Pergola, Boris Suchan

**Affiliations:** ^1^Department of Basic Medical Science, Neuroscience and Sense Organs, University of Bari ‘Aldo Moro’, Bari, Italy; ^2^Neuroscience Area, International School for Advanced Studies (SISSA), Trieste, Italy; ^3^Department of Neuropsychology, Ruhr-University Bochum, Bochum, Germany

**Keywords:** recognition memory, recollection, familiarity, recall, thalamus, retrosplenial cortex, prefrontal cortex, schizophrenia

## Abstract

Decades of research have established a model that includes the medial temporal lobe, and particularly the hippocampus, as a critical node for episodic memory. Neuroimaging and clinical studies have shown the involvement of additional cortical and subcortical regions. Among these areas, the thalamus, the retrosplenial cortex, and the prefrontal cortices have been consistently related to episodic memory performance. This article provides evidences that these areas are in different forms and degrees critical for human memory function rather than playing only an ancillary role. First we briefly summarize the functional architecture of the medial temporal lobe with respect to recognition memory and recall. We then focus on the clinical and neuroimaging evidence available on thalamo-prefrontal and thalamo-retrosplenial networks. The role of these networks in episodic memory has been considered secondary, partly because disruption of these areas does not always lead to severe impairments; to account for this evidence, we discuss methodological issues related to the investigation of these regions. We propose that these networks contribute differently to recognition memory and recall, and also that the memory stage of their contribution shows specificity to encoding or retrieval in recall tasks. We note that the same mechanisms may be in force when humans perform non-episodic tasks, e.g., semantic retrieval and mental time travel. Functional disturbance of these networks is related to cognitive impairments not only in neurological disorders, but also in psychiatric medical conditions, such as schizophrenia. Finally we discuss possible mechanisms for the contribution of these areas to memory, including regulation of oscillatory rhythms and long-term potentiation. We conclude that integrity of the thalamo-frontal and the thalamo-retrosplenial networks is necessary for the manifold features of episodic memory.

The only proof of there being retention is that recall actually takes place

(James, 1890).

Memory is a fascinating puzzle for neuroscientists: at first glance it seems straightforward to grasp the unity of this cognitive skill and its adaptive meaning; one is prompted to search for the storage room in the brain, like it happened in the beginning of memory research. Today there is consensus that many memory systems rely on dissociable neural substrates (Squire and Kandel, 2000). Within cognitive neuroscience, different research fields kept searching for a main character of the “memory play.” The best candidate for episodic memory, as defined by Tulving (1987, 2002) and Tulving and Markowitsch (1997, 1998), has been the hippocampus (HC^1^; Scoville and Milner, 1957).

Destruction of the HC is sufficient to wipe out novel episodic learning in humans and non-human primates (Mishkin, 1982; Zola-Morgan et al., 1982; reviewed by Aggleton and Brown, 1999). A less straightforward question is whether the loss of hippocampal function is necessary for episodic memory impairments. Damage to other regions, including the cortices of the parahippocampal gyrus, the prefrontal cortex (PFC), the thalamus, and the retrosplenial and posterior cingulate cortex (RSC), may also result in memory deficits (reviewed by Aggleton and Brown, 1999; Kopelman, 2002; Van der Werf et al., 2003a; Eichenbaum et al., 2007; Mitchell and Johnson, 2009; Vann et al., 2009a; Brown et al., 2010). All of these regions presumably perform operations that differ at least to some extent, so what we call “episodic memory” is the result of a number of sub-functions underlay by many brain regions.

A logical consequence is that damage to different brain areas leads to qualitatively different episodic memory impairments; this argument also applies to brain activations detected by means of neuroimaging techniques, which will overlap to a large extent, but not completely, depending on subtle task differences. One strategy to attack this complexity is to “average” the evidence and single out brain regions which regularly contribute to episodic memory. This way of proceeding is robust with respect to the inferences drawn, e.g., we can predict that surgical ablation of the HC will instantiate amnesia. However, this procedure is insufficient to study more fine-grained mechanisms underlying episodic memory, for example as a function of stimulus material or memory stage. This article reviews evidence on the involvement of other brain structures, aside from the HC, in what is the hallmark of HC-dependent memory: recall.

We will discuss clinical and neuroimaging literature about brain networks supporting different aspects of recall, particularly a thalamic-PFC and a thalamic-RSC network. A main tenet of this work is that interpretation of the results strictly depends on the tasks used to assess memory function. We will therefore argue that empirical work needs to study recall directly and to assess the neurophysiological correlates of recall subprocesses, in order to identify the mechanisms by which different brain regions contribute to recall. Damage to the thalamo-PFC and to the thalamo-RSC networks also impairs other cognitive functions beyond episodic memory, and hypotheses on the mechanisms of contribution of these networks to cognition are discussed in the final part of the review.

## The Main Actor – A Brief Reappraisal on the Role of the Medial Temporal Lobe in Recognition Memory and Recall

Since Mandler’s (1980) proposal to distinguish a form of recognition accompanied by retrieval of contextual and associative information (recollection) and one more implicit-like, simply consisting of the feeling that something is “old” or “new” (familiarity), the distinction between familiarity and recollection has been investigated widely in cognitive neuroscience. Increasing evidence has emerged in recent years supporting this “dual process model” of recognition memory (Yonelinas, 2002; Eichenbaum et al., 2007; Suchan et al., 2008; Ranganath, 2010; Voss and Paller, 2010). The dual process model assumes that the two processes are qualitatively different. Familiarity is graded and not well suited for associative memory; recollection accomplishes lively and detailed retrieval and is thought to be a threshold process. The main alternative view, the “single process account,” assumes a quantitative difference between recollection and familiarity, i.e., stronger memory traces elicit a feeling of recollection (Squire et al., 2007; Slotnick, 2013). Proponents of this view acknowledge that different behavioral outcomes indicate different neural processing within the MTL (Wixted et al., 2010). They stress, however, that tests commonly used introduce confounds in the form of different strength of the memory trace. In general, the techniques used to separately assess recollection and familiarity are debated; for a more complete picture of this controversy it is best to consult more focused reviews (Eichenbaum et al., 2007; Squire et al., 2007; Aggleton et al., 2010; Brown et al., 2010, 2012; Montaldi and Mayes, 2010; Wixted et al., 2010; Rugg et al., 2012; Slotnick, 2013).

**Figure 1:**
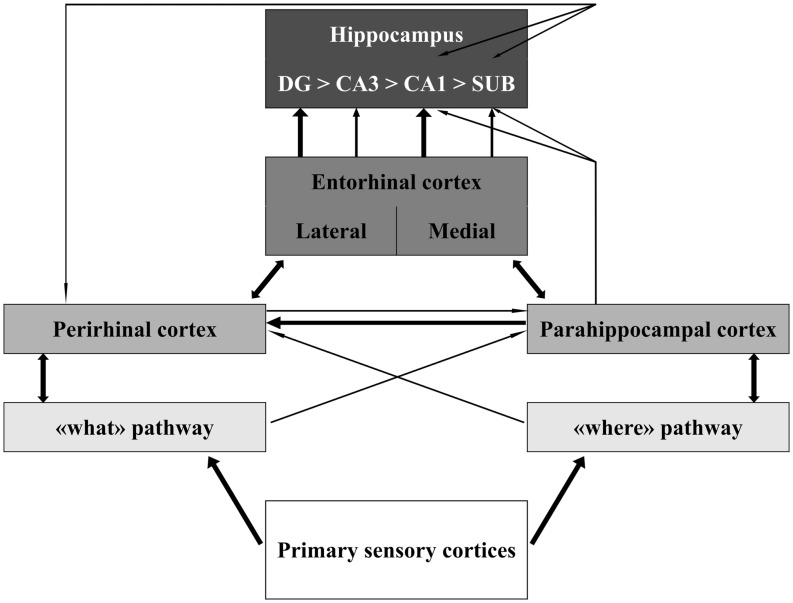
**Schematic representation of the organization of the medial temporal lobe in information processing**. The thickness of the lines represents the weight of the connections. Notice that sensory information is only partly integrated at the level of the perirhinal, parahippocampal, and entorhinal cortices. Information about different aspects of the sensory stimulus converges in the hippocampus through largely segregated pathways. Modified from Aggleton (2012). Abbreviations: DG, dentate gyrus; CA, cornus ammonis; SUB, subiculum.

The MTL includes the HC and the parahippocampal cortices. The neocortical areas that send inputs into and receive outputs from the MTL include all higher order “association” areas and no primary sensory (except for the olfactory) or motor cortices. This connectivity pattern is illustrated in Figure 1. The HC is on top of this information flow (Lavenex and Amaral, 2000; Witter et al., 2000; Eichenbaum and Lipton, 2008). The outputs of hippocampal processing are directed back down this information path in reversed order.

It is debated whether the distinct processes subserved by the HC and the parahippocampal cortices correspond to recollection and familiarity (Yonelinas et al., 1998; Holdstock et al., 2002, 2005; Mayes et al., 2002; Yonelinas, 2002; Davachi et al., 2003; Bastin et al., 2004; Ranganath et al., 2004; Aggleton et al., 2005; Uncapher and Rugg, 2005a,b; Montaldi et al., 2006; Uncapher et al., 2006). There is agreement, however, that associative memory encoding tasks recruit the HC, relative to tasks without requirement (or success) of associative encoding (Henke et al., 1997, 1999; Sperling et al., 2003; Achim and Lepage, 2005; Chua et al., 2007). The neural substrates of recognition memory also depend on more subtle differences related to stimuli presentation (Henderson et al., 2003; Cipolotti et al., 2006; Bird et al., 2007; Peters et al., 2007a,b; Awipi and Davachi, 2008). Of interest to the empirical setting of novel investigations, there is consensus that recall and recognition are dissociable cognitive skills, and that the HC is necessary for recall, but likely insufficient. Yonelinas et al. (2010), for instance, highlighted the role of the PFC in recognition memory, concluding that the extant evidence favors a prefrontal contribution to recollection, in particular during encoding.

Thus, the constituents of the MTL play different roles in recognition memory and recall, with a major role of the HC in recall. The contribution of other areas of the MTL to recognition memory is intensely debated. Beyond the MTL, there is evidence on the involvement of other brain areas in recall. This will be the subject of Section “A Crowded Stage – Evidence for Other Recognition and Recall Networks.”

## Intricated Plots – How to Assess the Contribution of Thalamo-Cortical Networks to Memory

Since this review focuses on clinical and neuroimaging evidence, we will briefly discuss the impact of testing procedures on results derived by these techniques.

Recognition memory tasks fall into two major categories: subjective and objective (Eichenbaum et al., 2007). Subjective tasks rely on self-assessment of memory traces, and include for instance the remember/know paradigm (Tulving, 1987) and the receiving operating curves based on confidence levels (Yonelinas, 2002). Objective paradigms, instead, test directly for memory of associations related to the recognition cue; source memory and recall tests are examples of this kind of experimental procedure.

In remember/know paradigms subjects are instructed to assess whether their memory is more based on conscious associations or on “feelings” of familiarity, entailing the sensation that one “knows” an item but does not “remember” anything about it. These paradigms have been criticized because they may rather tap subjective awareness of one’s memory than “true” features of the memory trace (Gardiner, 1988; Newell and Dunn, 2008; Geraci et al., 2009; McCabe and Geraci, 2009). Moreover, memory strength might confound remember/know results (Slotnick, 2013).

Receiving operating curves, on the other hand, cannot discriminate between “recollected” and “recognized” stimuli, but provide a global estimation of familiarity and recollection in one condition, individual, or group. The estimates are based on the notion that recollection is a threshold process (Yonelinas et al., 2010), an assumption that did not fail to trigger criticism (Wixted et al., 2010; Slotnick, 2013), although recent fMRI evidence appears to support it (Pustina et al., 2012). On the other hand, the idea that lower confidence involves greater familiarity is still prone to the alternative interpretation that recollection simply reflects greater memory strength (Wixted, 2007).

Montaldi et al. (2006) and Kafkas and Montaldi (2012) developed a subjective task that, like Remember/Know, entails training subjects to distinguish recollection from familiarity. Participants focus on familiarity and assess their confidence (from one to three), while recollection should be avoided. If a participant detects that he/she has been using recollection, he/she reports this. This task effectively matches memory strength, with the caveat that subjects are focusing on familiarity: in objective tasks they are usually actively engaged in recall. This novel subjective task appears to be a promising tool to study familiarity free of the “memory strength” confound. Results obtained on “recollection” trials by using this task, however, share the general limitations of subjective tasks and introduce the additional feature that recollection is undesired. Participants’ orientation at encoding and retrieval may be different from what is typical in objective tasks.

Objective paradigms are more powerful than subjective paradigms in indicating recollection. In particular, objective paradigms allow trial-by-trial discrimination of recollection by asking subjects to report features associated with the recognition cue, which may be perceptual (e.g., color; Cycowicz et al., 2001), contextual (e.g., place where the cue was previously shown; Cansino et al., 2002), semantic (e.g., match or mismatch with the category of other items; Pergola et al., 2013b), and episodic (e.g., decision taken during previous exposure or imagination of the items; Vilberg and Rugg, 2012). There is general agreement that, when memory strength and confidence are equated, recollection is more likely to be involved in recognition memory followed by recall, as compared to familiarity (Brown et al., 2010; Wixted et al., 2010).

Nevertheless, objective paradigms present a certain degree of variability, and subtle details can change the pattern of activations in a neuroimaging study, as well as the pattern of deficits displayed by clinical samples. For example, it has been shown that some forced choice tasks also entail neural correlates typical of familiarity (Quamme et al., 2007; Diana et al., 2008). Mayes et al. (2007) proposed that this might happen because of lower-level relational operations performed in the parahippocampal and perirhinal cortices. Items belonging to the same context (e.g., steer and brakes in a car) might be encoded already at the level of the perirhinal cortex: overlearned associations require less integration. This argument extends to material learned through “unitized” representation (e.g., the word association sun-set compared to sun-toy). Moreover, remembering the information associated with a recognition cue when choosing between two and three possibilities is prone to guessing influence. Recall tasks which require retrieval of a unique association, instead, are robust with respect to guesses and to familiarity (Montaldi and Mayes, 2010), a strategy recently used in both clinical and neuroimaging setting (Pergola et al., 2012, 2013b,d). The trials in which correct recall occurs are most likely recollection trials, although the converse is not true: it is possible that a participant recognizes the cue based on recall, but not on recall of the information tested, e.g., an associated picture or context (non-criterial recollection: see Yonelinas et al., 2010).

In our opinion, investigations of the recollection/familiarity dichotomy are made difficult by the pragmatic definitions of recollection and familiarity. Both are thought to support recognition, and additionally recollection is thought to support recall. Hence evidence of recall is commonly used to infer recollection; lack of recall is used to infer familiarity (see for example Slotnick, 2013). However, in order to show the involvement of a brain structure in familiarity processing, it is necessary to find exclusive correlates of familiarity – something that recollection *cannot* support. It is our impression that consensus on such specialized features of familiarity has not yet been reached. Putative correlates of familiarity that have been disputed include reaction times, responses given under time pressure, differential modulation of responses induced by perceptual manipulation, and electrophysiological components (Eichenbaum et al., 2007; Squire et al., 2007; Brown et al., 2010, 2012; Montaldi and Mayes, 2010; Wixted et al., 2010; Paller et al., 2012; Rugg et al., 2012). The paradigms that seem most successful in detecting familiarity are subjective tasks (Yonelinas, 2002; Montaldi et al., 2006), within the limitations discussed. Hypotheses about the involvement of select brain structures in familiarity appear empirically ill-posed, if they must rely on lack of evidence of recollection.

Following this framework, in the following we will differentiate *recall* (i.e., recognition memory followed by recall of contextual or associative details) and *recognition without recall* (i.e., correct judgment of previous occurrence not followed by successful retrieval of contextual and associative information). These behavioral outcomes are easier to probe empirically than the putative underlying processes (recollection and familiarity). It is important for the following of the article to keep in mind this distinction between behavior and underlying processes.

## A Crowded Stage – Evidence for Other Recognition and Recall Networks

The earliest observations on non-hippocampal based amnesia relate to the “Wernicke–Korsakoff syndrome,” a degenerative disease caused by depletion of thiamine (often secondary to alcohol abuse; for reviews see Kopelman, 2002; Kopelman et al., 2009). Amnesic symptoms in Korsakoff patients have been related to damage in the mammillary bodies and the anterior nuclei of the thalamus (abbreviated in the following as AT; Victor et al., 1989; Harding et al., 2000). Another clinical condition leading to amnesia is thalamic stroke (reviewed by Schmahmann, 2003; Carlesimo et al., 2011). Behavioral symptoms include anterograde amnesia, executive deficits, and rarely retrograde memory loss. Implicit memory is mostly preserved, much like in hippocampal amnesia (Daum and Ackermann, 1994; but see Exner et al., 2001).

Aggleton and Brown (1999) challenged the distinction between medial temporal and subcortical amnesias by proposing that the AT are functionally linked to the HC (i.e., critical for recollection), while the mediodorsal nucleus (MD) contributes more to familiarity based on its connections to the perirhinal cortex. This model has been recently revised (see Aggleton et al., 2011, for an update). We will now evaluate the extant evidence on the role of the MD and the AT in recognition memory in the framework of the thalamo-PFC and the thalamo-RSC networks.

### The thalamo-prefrontal network

**Figure 2:**
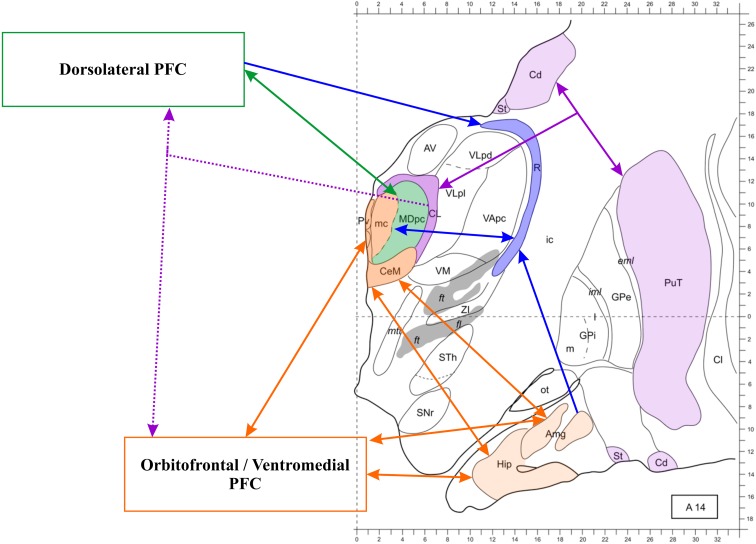
**The thalamo-prefrontal cortical network**. Transversal section 14 mm anterior to the posterior commissura. The thalamus and the prefrontal cortex (PFC) are connected through partly independent pathways. Dotted lines represent widespread projections. Notice the triangular connections involving the medial temporal lobe, the midline thalamus and the orbitofrontal, and ventromedial cortex. The mediodorsal nucleus (MD) is involved in multiple pathways, also including the reticular nucleus (R), which receives projections from the PFC and the amygdala as well as reciprocal connections to the MD (both subunits). The MDmc, which is represented in the same color as the midline nuclei because they present functional commonalities, is not connected to the hippocampus, but it receives amygdalar projections and is reciprocally connected to the orbitofrontal and ventromedial PFC. The intralaminar nuclei (only the centrolateral nucleus (CL) is represented in this section) are part of the thalamo-striato-frontal network (striato-frontal connections are not represented). Modified from Morel (2007). Abbreviations: Amg, amygdala; AV, anteroventral nucleus; Cd, caudate nucleus; Cl, claustrum; eml, external medullary lamina; ft, fasciculus thalamicus; ic, internal capsula; iml, internal medullary lamina; GPe, globus pallidum, pars externa; GPi, globus pallidum, pars interna; Hip, hippocampus; MDpc, parvocellular MD; mc, magnocellular MD; mtt, mammillothalamic tract; ot, olfactory tubercle; PuT, putamen; Pv, paraventricular nucleus; SNr, substantia nigra, pars reticulate; St, striatum; STh, subthalamic nucleus; VApc, ventral anterior nucleus, parvocellular portion; VLpd, ventrolateral nucleus, posterior dorsal subunit; VLpl, posterior lateral subunit; VM, ventromedial nucleus; ZI, zona incerta.

The conspicuous evidence delineating the anatomical basis of the thalamic-PFC network has already been discussed elsewhere (Taber et al., 2004; Byne et al., 2009; Klein et al., 2010; Barbas et al., 2012). Figure 2 illustrates the main patterns of anatomical connection of this network.

At least three important circuits involved in episodic memory relate the thalamus and the PFC. The first is the reciprocal MD-PFC connection, which shows corresponding thalamic medio-lateral and prefrontal ventromedial-dorsolateral topographical gradients (Russchen et al., 1987; Barbas et al., 1991; Ray and Price, 1993). In other words, the MD-PFC connectivity is not homogeneous within the nucleus. In humans, the MD^2^ is comprised of a magnocellular portion (MDmc), covering the medial third of it, and a parvocellular portion (MDpc), larger and lateral to the MDmc. The connectivity patterns of the MDmc and the MDpc differ (for discussion see Barbas et al., 2012; Pergola et al., 2012; Mitchell and Chakraborty, 2013). The MDmc is reciprocally connected to the ventromedial PFC and also receives afferents from the MTL (Aggleton, 2012). The MDpc, instead, is reciprocally connected to the dorsolateral PFC (DLPFC) and this is its major source of input, although it receives further input from other prefrontal areas (Mitchell and Chakraborty, 2013). There is no evidence of input from the MTL to the MDpc (Mitchell and Chakraborty, 2013). Byne et al. (2009) observed that, similar to the MDmc, also the medial pulvinar is connected to the PFC as well as with temporal and parietal cortices, and may be involved in declarative memory (see also Nadeau and Crosson, 1997). This suggestion has been supported by more recent neuroimaging findings on pulvinar activations during associative memory encoding (Pergola et al., 2013b).

The second pathway includes the diffuse projections from the intralaminar nuclei (ILN)^3^ to the PFC. While the ILN send specific projections to the basal ganglia (Preuss and Goldman-Rakic, 1987; Barbas et al., 1991; Sadikot et al., 1992), projections to the PFC are more sparse and widespread. The functional role of these connections is unclear. The ILN are considered part of a cerebellar-striato-frontal network that has been proposed to be essential for language production and control (Nadeau and Crosson, 1997). It has also been proposed that the activity of these nuclei may rapidly recruit large cortical portions and entrain synchronization of cortical activity (reviewed by Jones, 2007), hence contributing to allocate attentional resources (Van der Werf et al., 2002).

A third pathway, proposed to especially contribute to attention control, includes the reticular thalamic nucleus (RTN), the main source of GABAergic input to the thalamus. The thalamo-cortical cells of the AT, MD, and other nuclei are mostly glutamatergic and excite cortical neurons; local GABAergic interneurons, however, constitute up to 25–30% of cells, a peculiarity of the primate thalamus, compared to the rodent thalamus (reviewed by Jones, 2007). Beyond this “intrinsic” inhibition, thalamic nuclei are regulated by the RTN, which receives collaterals from both thalamo-cortical and cortico-thalamic fibers, but only inhibits thalamic cells (Avanzini et al., 1996). Therefore this nucleus is in the place to switch between patterns of thalamic electrophysiological activity, by selectively inhibiting the thalamo-cortical projections (Crick, 1984). Notably, the left and right RTN are connected directly, unlike most nuclei of the dorsal thalamus. The topographical order of the connections between the RTN and the nuclei of the dorsal thalamus forms “sectors” of the RTN that selectively control specific circuits and functions, thus affecting specific cortical areas (Barbas et al., 2012). On the other hand, the activity of an RTN subregion can quickly recruit the whole nucleus, and thereby the whole thalamo-cortical network, through gap junctions (Wang and Rinzel, 1993). Intriguingly, the MD interacts in a specific way with the RTN. All other nuclei project to a specific sector of the RTN, whereas the MD projects to all RTN subregions (Barbas et al., 2012). The same holds for the PFC, which regulates RTN activity as a whole (Zikopoulos and Barbas, 2006). This anatomical evidence suggests that the MD-PFC-RTN circuitry is critical for allocating cognitive resources and that the MD-PFC interactions are able to effectively modulate the activity in other thalamic areas through the interaction with the RTN. In sum, anatomical evidence differentiates three integrated components of the thalamo-PFC network: the MD-PFC connections, further composed of two pathways (magnocellular and parvocellular) which project to distinct cortical areas; the ILN-PFC connections; and the RTN-PFC connection, which also interacts with the MD.

Lesion studies in animals highlighted the importance of this MD-PFC system for episodic memory (see Mitchell and Chakraborty, 2013 for review). However, it has been suggested that deficits found in rewarded recognition tasks may reflect the effects of MD damage on aspects of task performance other than recognition, particularly on reward association learning (Corbit et al., 2003; Cross et al., 2012; see Baxter, 2013 for a discussion based on evidence from non-human primates). There might be differences between rodents and primates as concerns the role of the MD-PFC network in recognition memory, reflecting a greater influence of PFC-dependant processing in object recognition in primates (Aggleton et al., 2011; Cross et al., 2012). This notion can help reconcile seemingly contrasting findings in animals and humans. The MD nuclei of rodents and primates differ in the relative dimensions of their subunits, in the expression of intrinsic GABAergic neurons (reviewed by Jones, 2007), in the connectivity to the RTN (Zikopoulos and Barbas, 2012), in the expression of dopaminergic receptors (Garcia-Cabezas et al., 2007, 2009) and of transcripts related to dopaminergic transmission (Hurd and Fagergren, 2000). So many differences entangle inferences on the functions of the human thalamo-PFC network based on work with rodents.

The evidence available based on studies with non-human primates confirms the role of this network in learning and memory, especially with respect to an involvement of the MDmc in encoding (reviewed by Baxter, 2013). Even though the concerns about mixed influences of episodic memory and reward processing still apply, work with animal models highlights multiple interactions between the thalamus and the PFC, with the MD being a key hub of the network.

#### Clinical evidence

Patients with frontal lobe lesions are impaired in recognition and recall, with disproportionate impairment on the latter (Shimamura, 1995; Wheeler et al., 1995). These patients have difficulties with strategic aspects of recall, i.e., effectively generating and using cues to build/retrieve memory traces. The PFC is ubiquitously activated in recognition memory fMRI experiments (Cansino et al., 2002; Dobbins and Wagner, 2005; see Mitchell and Johnson, 2009 for a review), yet its exact contribution is far from clear. In event-related potentials (ERP) studies, frontal activity is found during episodic memory encoding (Neufang et al., 2006; Blumenfeld and Ranganath, 2007; Kim et al., 2009; Pergola et al., 2013d), as well as retrieval (Allan and Rugg, 1998; Duzel et al., 1999; Ranganath et al., 2000; Badgaiyan et al., 2002; Dobbins et al., 2002; Rugg and Curran, 2007; Pergola et al., 2013d). Most likely, the pattern of activations found in recognition memory studies at frontal sites actually depends on the activity of several PFC subregions processing novelty detection, relational encoding, maintenance, weighing, and selection of concurrent responses (Thompson-Schill et al., 1997; Dobbins and Han, 2006; Blumenfeld and Ranganath, 2007; Burgess et al., 2007; Bergstrom et al., 2013). For example, the ventrolateral PFC seems involved in memory formation irrespective of its associative nature (Blumenfeld and Ranganath, 2007; Mitchell and Johnson, 2009), while the DLPFC specifically contributes to successful associative encoding (Dolan and Fletcher, 1997; Staresina and Davachi, 2006; Murray and Ranganath, 2007; Mitchell and Johnson, 2009; Blumenfeld et al., 2011; Huijbers et al., 2013).

As regards the thalamus, deficits of recall and associative memory have been documented following ischemic lesion in the territory of the MD (Zoppelt et al., 2003; Edelstyn et al., 2006, 2012a; Soei et al., 2008), although those studies could not rule out a role of damage to the mammillothalamic tract (MTT) in the deficit pattern (discussed by Carlesimo et al., 2011; the MTT is considered part of the thalamo-RSC network). We performed a systematic review^4^ of all case reports of thalamic stroke *with damage in the territory of the MD and without apparent damage in the territory of the AT and the MTT*. Only reports including neuropsychological assessment of memory skills were included. Results are shown in Table 1.

**Table 1:** **Clinical literature on cases with lesion to the mediodorsal thalamic nucleus**.

I. Article	II. Laterality	III. Phase of lesion at test	IV. MTT involvement	V. Medial MD lesion	VI. Lateral MD lesion	VII. Recall	VIII. Recognition	IX. Encoding deficits	X. Retrieval deficits	XI. Comments
Speedie and Heilman (1982)	L	Acute, sub-acute	*Perhaps*	Unclear	Unclear	Impaired	NA	–	Apparent deficit only after delay	CT scan suggests that *the MTT could be involved. Psychiatric history*
Winocur et al. (1984)	B	Chronic (<1 Y)	*Perhaps*	Yes	Unclear	Impaired	Impaired	Greater deficit after short stimuli presentation	Deficit more evident after delay	CT scan suggests that *the MTT could be involved*. Deficit interpreted as encoding-dependent
von Cramon et al. (1985)^a^	L (P6); B (P5)	Chronic (>1 Y)	NO	Minor	Yes, not associated with amnesia	Spared	Spared	–	–	Overlap analysis associated amnesia with MTT damage
Bogousslavsky et al. (1986)	L	Acute	NO	Yes	Yes	Impaired	NA	Short-term memory deficits	–	Post-mortem confirmation. ILN and *extrathalamic damage*
Kritchevsky et al. (1987)	R (P1); B (P2)	Sub-acute	NO	Yes	Only in P2	Spared	Spared	–	–	<15% of MD volume involved
Reilly et al. (1992)	B	Acute, chronic (<1 Y)	*Unclear*	Unclear	Unclear	Amnesia (two cases)	NA	Short-term memory deficits	–	Limited neuropsychological examination
Calabrese et al. (1993)	B	Sub-acute	NO	Yes	Minor	Impaired	Impaired	Immediate recall deficits	Deficit more evident after delay	
Shuren et al. (1997)	R	Acute, chronic (<1 Y)	NO	Yes	Yes	Spared but source memory deficit	Spared	–		ILN damaged. Source memory for temporal order was selectively impaired
Isaac et al. (1998)	B	Chronic (>1 Y)	NO	Yes	Yes	Impaired	Impaired	–	Deficit more evident after delay	*Atrophy of the mammillary bodies*
Fukutake et al. (2002)	L	Sub-acute	NO	Yes	Minor	Spared	NA	–	–	*Test under pharmacological treatment*. Limited neuropsychological examination
Van der Werf et al. (2003b)^b^	L	Chronic (>1 Y)	NO	NO	Yes	Mildly impaired	Mildly impaired	–	Increased forgetting rate	Impaired visual memory. P13 showed impaired verbal memory and *extrathalamic lesions*
Zoppelt et al. (2003)	L (five cases), R (four cases)	Chronic (mostly >1 Y)	*Perhaps*	Two cases, familiarity and recollection deficit	Three cases, recollection deficit	Impaired	Impaired in some cases	Backward span impaired, short-term memory spared	Increased forgetting rate	*Damage to the MTT* was likely in several cases
Tanji et al. (2003)	L	Chronic (<1 Y)	NO	Yes	Minor	Impaired	Mildly impaired	Immediate recall deficits	Deficit more evident after delay	*Extrathalamic damage*
Soei et al. (2008)	L (six cases); R (three cases); B (one case)	Chronic (mostly >1 Y)	*Perhaps*	Non-associative memory impaired	Milder deficit	Impaired	Impaired in some cases	–	Apparent deficit only after delay	*Damage to the MTT* was likely in several cases
Hampstead and Koffler (2009)	B	Sub-acute	*Perhaps*	Yes	Yes	Impaired	Spared	Immediate recall deficits	Deficit more evident after delay	Profound recall deficit. *History of substance abuse. Extrathalamic damage*
Ioannidis et al. (2012)	B	Chronic (>1 Y)	*Unclear*	Yes	Yes	Impaired	NA	Immediate recall deficits	–	Profound recall deficit. Neuroimaging data suggest that *the MTT might have been affected*
Pergola et al. (2012)^c^	L (two cases); R (four cases); B (two cases)	Chronic (>1 Y)	NO	Yes	Yes	Impairment proportional to MDpc damage	Impaired in some cases	Immediate recall deficits	Deficit more evident after delay	Relatively mild deficits, with patients mostly between 0 and 3 standard deviations below controls

*Only studies in which authors did not find clear evidence of damage to the anterior nuclei (including the laterodorsal nucleus) and to the mammillothalamic tract were included. Shaded rows highlight studies in which confounding factors should be considered when evaluating results. Confounding factors are written in italic font. Acute phase: ≤1 week after lesion onset; sub-acute phase: 1 week < lesion-test interval ≤2 months; chronic phase: >2 months after lesion onset*.

*B, bilateral lesion; ILN, intralaminar thalamic nuclei; L, left-sided lesion; MD, mediodorsal thalamic nucleus; MTT, mammillothalamic tract; NA, not assessed; P, patient; R, right-sided lesion; Y, year*.

*^a^ Two cases without AT/MTT damage considered (Patients 5 and 6)*.

*^b^ Two cases without AT/MTT damage considered (Patients 1 and 13)*.

*^c^ Eight cases without AT/MTT damage were considered (Patients 2, 3, 5, 6, 7, 8, 9, 16). All patients except for P7, P8, and P9 were free of psychoactive medication*.

We considered 17 studies, for a total of 44 cases. A first look at Table 1 reveals how heterogeneous the cases were with respect to laterality, lesion-test interval, and lesion assessment. The paucity of studies meeting the requirements we set and their heterogeneity aligns with the current lack of agreement on the function of the MD.

This analysis reveals that no single report documents impairments of recognition without recall deficits (Table 1, columns VII and VIII), a fact also acknowledged by other researchers (Aggleton et al., 2011; Carlesimo et al., 2011; Mitchell and Chakraborty, 2013). The general pattern of deficits is consistent with the idea that a primary impairment on recall entails a deficit in recognition memory because of disrupted recollection (Pergola et al., 2012).

The picture becomes more complicated when the evidence is evaluated more strictly. In several studies (Table 2, gray background) lesion to the MTT or to extrathalamic regions cannot be excluded; in others, pharmacological treatment or history of psychiatric disorders and/or substance abuse limit the clarity of the results. When studies with these potential confounds are excluded, only 13 cases remain (von Cramon et al., 1985; Kritchevsky et al., 1987; Calabrese et al., 1993; Shuren et al., 1997; Van der Werf et al., 2003b; Pergola et al., 2012). Two observations can be made on these studies: first, these most informative reports document less severe deficits; second, it seems that more recent reports found greater impairments compared to the earlier ones. We suggest that more recent studies employed more sensitive and/or extensive testing, changing the framework from the study of “amnesia” to the study of specific memory deficits.

**Table 2:** **Clinical literature on cases with lesion to the anterior thalamic nuclei**.

I. Article	II. Laterality	III. Phase of lesion at test	IV. MTT involvement	V. Recall	VI. Recognition	VII. Encoding deficits	VIII. Retrieval deficits	IX. Comments
Hankey and Stewart-Wynne (1988)	L	Acute	YES	Impaired	NA	Immediate recall deficits	–	Post-mortem confirmation of lesion localization
Ott and Saver (1993)^a^	L	Acute and chronic (<1 Y)	Perhaps	Impaired	NA	–	Apparent deficit only after delay	Follow-up after 5 months from onset revealed an *additional lesion in the caudate nucleus. MD could have been affected*
Clarke et al. (1994)	L	Acute and chronic (<1 Y)	YES	Impaired in the verbal domain	Impaired in the verbal domain	–	Short-term memory was spared	Sensitivity to interference. ILN clearly involved. Posterior cingulate cortex showed hypometabolism
Hanley et al. (1994, 2001)^b^	L	Chronic (>1 Y)	YES	Impaired	Spared	–	Deficit interpreted as retrieval-dependent	*Involvement of the fornix and the caudate nucleus*
Ghika-Schmid and Bogousslavsky (2000)	L (eight cases), R (four cases)	Acute, sub-acute, and chronic (<1 Y)	YES	Impaired	Mildly impaired	Immediate recall deficits in the acute phase	Deficit more evident after delay. Repeated exposure did not improve performance. Confabulations, intrusions, false memory during the acute and sub-acute phases	Palipsychism in the acute phase, sensitivity to interference, word finding difficulties

*Only studies in which no clear evidence of damage to the mediodorsal nucleus was found were included. Shaded rows highlight studies in which confounding factors should be considered when evaluating results. Confounding factors are written in italic font. Acute phase: ≤1 week after lesion onset; sub-acute phase: 1 week < lesion-test interval ≤2 months; chronic phase: >2 months after lesion onset*.

*ILN, intralaminar thalamic nuclei; L, left-sided lesion; MD, mediodorsal thalamic nucleus; MTT, mammillothalamic tract; NA, not assessed; P, patient; R, right-sided lesion; Y, year*.

*^a^ One case with anterior thalamic lesion was considered*.

*^b^ The lesion was consequent to rupture of an aneurysm*.

Pergola et al. (2012), for instance, found a decline in recall performance in patients with focal medial thalamic stroke, who were not impaired in recognition without recall. The task involved single-item recognition and cued recall of uniquely paired associates. Quantitative assessment of the lesions revealed that patients’ recall deficits were proportional to damage to the MDpc (but not MDmc/midline or ILN). Even though some of the patients showed evidence of lesion in the MTT, those with no evidence of such damage showed a deficit pattern similar to that of the whole sample. Overlap/subtraction analysis confirmed that in these patients memory deficits were associated to lesion in the MDpc.

This evidence suggests that the MDmc and the MDpc might contribute differently to recognition memory. Zoppelt et al. (2003) proposed that the MDmc could be related to familiarity. Conversely, the recall deficits observed by Pergola et al. (2012) selectively involved the MDpc in recall performance. It can thus be hypothesized that the MDpc is required for recall, while the MDmc is required for recognition without recall. Clinical evidence appears inconclusive in this respect (Table 1, columns V and VI), and data from animal studies are problematic for such a proposal (Mitchell and Chakraborty, 2013). Likewise, clinical evidence is of little avail with respect to the contribution of the MD to the encoding or retrieval phase of memory processing (Table 1, columns IX and X; Winocur et al., 1984; Mitchell and Chakraborty, 2013).

#### Neuroimaging evidence

Neuroimaging evidence specifically addressing the role of the thalamus in cognition is sparse (Metzger et al., 2013). To our knowledge, evidence on the differential contribution of the two portions of the MD to episodic memory is limited to a single fMRI study (Pergola et al., 2013b). The study employed a single-item recognition and associative cued recall task and included an anatomical parcellation of the functional clusters activated during task performance. Robust thalamic activation characterized recognition accompanied by recall, compared to recognition not followed by recall, consistent with Achim and Lepage (2005), who found higher thalamic activation during associative than single-item recognition. The MDpc was activated during both encoding and retrieval, similarly to the DLPFC; critically, the MDpc was more activated during recall than during recognition not followed by recall, matching the clinical results and supporting the hypothesis that an MDpc-DLPFC network subserves recall. Activation in the MDmc was only found during retrieval. No thalamic voxel displayed greater activation during recognition without recall compared with recognition followed by recall.

These findings leave unexplained whether the MDmc preferentially supports recognition without recall rather than recall. Perhaps support to this hypothesis comes from two studies performed by Montaldi et al. (2006) and Kafkas and Montaldi (2012), using the task previously described (see Intricated Plots – How to Assess the Contribution of Thalamo-Cortical Networks to Memory). Montaldi et al. (2006) found that activity in the dorsomedian thalamus correlated with familiarity self-reported confidence, although the same region was equally activated during familiarity based and recollection-based judgments. Kafkas and Montaldi (2012) reported greater dorsomedian thalamic activity during high-confidence familiarity trials than during unwanted recollection trials. The same pattern was observed in the orbitofrontal cortex, while the DLPFC and more lateral thalamic clusters were significantly more activated during recollection than familiarity. Intriguingly, the thalamic cluster activated by familiarity was remarkably medial, with the peak located in a position consistent with the putative MDmc or midline territory (coordinates in MNI space: *x* = −1; *y* = −15; *z* = 6). The FMRIB connectivity atlas (Behrens et al., 2003; Johansen-Berg et al., 2005; http://www2.fmrib.ox.ac.uk/connect/) reports the following projection probabilities at these coordinates: sensory cortex 0.02; Occipital cortex 0.10; PFC 0.02; temporal cortex 0.24. This connectivity pattern is compatible with localization of this peak in the MDmc, the midline nuclei, or the AT, because of the relatively high probability of connection with the temporal lobe, but very unlikely in the MDpc. Kafkas and Montaldi (2012) stressed that the dorsomedian thalamic cluster did discriminate hits from misses also in the recollection condition and that, in general, clusters activated during familiarity included subsets of voxels of clusters activated during recollection.

The findings by Kafkas and Montaldi (2012) and Pergola et al. (2013b) agree to some extent. Both studies observed higher thalamic and PFC activation during recall than recognition without recall and an involvement of the MD and the DLPFC in recollection-based recognition, in accord with other fMRI studies (Mottaghy et al., 1999; de Rover et al., 2008; Blumenfeld et al., 2011). On the other hand, the results by Montaldi’s group support a role of the MD in familiarity-based recognition, whereas our results suggest that the MDpc is specifically activated by recognition followed by recall. The implications of this discrepancy are discussed in the next section.

#### Models on the functional architecture of the thalamo-PFC network

Clinical results fail to support the hypothesis of selective familiarity deficits following lesion to the MD. This lack of evidence led Aggleton et al. (2011) to revise their 1999 model. In their multi-effect multi-nuclei (MEMN) model of thalamic contribution to recognition memory, they proposed that the MD, the ILN, and midline nuclei, as well as the pulvinar, may contribute in a graded way to both recollection and familiarity. It was proposed that the MD contributes more to familiarity than to recollection. In light of the data reviewed above, support for the proposal that the MD contributes to familiarity remains shaky, possibly limited to the fMRI results obtained by Montaldi et al. (2006) and Kafkas and Montaldi (2012).

In our opinion there are two possibilities to reconcile the seemingly conflicting neuroimaging findings obtained by Montaldi et al. (2006) and Pergola et al. (2013b). Firstly, the MDpc may support recall, while the MDmc may support familiarity. The distinction between the MDmc and MDpc connectivity patterns has also been advocated by Aggleton (2012) as a discriminant in their functional role; in this perspective, the finding by Kafkas and Montaldi (2012) that a region in the orbitofrontal cortex responded more strongly to familiarity than recollection avails the dissociation between the MDmc and the MDpc, because the MDmc is more strongly connected to the orbitofrontal cortex compared to the MDpc. The dichotomy between the magnocellular and the parvocellular MD may encompass a wider ground than the recollection/familiarity distinction, since the MDmc has also been implicated in reward-based learning. Lesions to the MDmc impair reward-based learning in rodents (Mitchell and Dalrymple-Alford, 2005), and also in monkeys, in particular during acquisition (i.e., initial learning; Mitchell and Gaffan, 2008). This impairment is also seen after disconnection from the ventromedial PFC (Mitchell et al., 2007), which suggests that it does not depend on the MTL afferents. Removal of cortical neurons produces a greater impairment in memory retrieval than in new learning, whereas subcortical damage produces a greater impairment in new learning than in memory retrieval (Mitchell et al., 2008). Although intriguing, a stark cognitive dissociation between subdivisions of the MD is weakly supported by lesion evidence in animals overall (Mitchell and Chakraborty, 2013). The parcellation of the MD needs further investigation in humans, and should in our opinion be taken into account in future clinical and neuroimaging studies. It is especially important to quantify the extent of the lesions and activations detected to bridge the gap with evidence based on animal studies. Even though the deficits found by Pergola et al. (2012) were relatively mild, quantitative assessments of the lesions revealed that the maximal volume loss in the MDpc was <30% and in most cases unilateral. This percentage is very far from the complete removal of select nuclei that is accomplished with the use of animal models, and it is possible that the role of the MD in memory is underestimated because of the limited extent of the lesions available for study.

A second possibility is that the difference between the studies lies in the tasks used. In particular, the instructions of the task employed by Montaldi et al. (2006) and Kafkas and Montaldi (2012) induced participants to focus their attention on the detection of familiarity during the retrieval phase. This focus on familiarity during retrieval could modulate the cognitive orientation of participants during encoding. The instructions of the task employed by Pergola et al. (2013b), instead, focused attention on associative recall. What kind of memory system is one that changes behavior depending on the conditions set up by the experimenter? The answer is, perhaps, that the MD-PFC network responds to cognitive orientation by setting its function depending on the behavioral goal (Monchi et al., 2001). This hypothesis is consistent with current models of the functional role of the DLPFC (Dobbins and Han, 2006). Accordingly, activation of the thalamo-PFC network is observed not only during retrieval, but also during encoding (Blumenfeld et al., 2011; Pergola et al., 2013b). Following this interpretation, damage to the MD (perhaps the MDpc in particular) would be expected to disrupt goal-directed memory processing more than goal-unrelated memory. This hypothesis remains to be tested.

The functions of the thalamo-PFC network seemingly encompass a wider cognitive domain than episodic memory (see Metzger et al., 2013, for a review). Patients with ischemia in the medial thalamus manifest a spectrum of symptoms including distractibility, aphasia, irritability, disinhibiting, disorganization of perception and thoughts, and executive deficits (Nadeau and Crosson, 1997; Schmahmann, 2003; Van der Werf et al., 2003a; Carrera and Bogousslavsky, 2006; Peterburs et al., 2011; Edelstyn et al., 2012b; Biesbroek et al., 2013). Ischemic lesions in the left medial thalamus, affecting the MD and the ILN, can result in semantic memory deficits in non-aphasic patients (Pergola et al., 2013a). Patients show deficits on a semantic retrieval task requiring activation of a third object from a pair of cues (Kraut et al., 2002b, 2006, 2007; see also Kraut et al., 2002a; Segal et al., 2003; Assaf et al., 2006). As it can be expected, the PFC also plays a role in semantic memory, together with the lateral temporal lobe, the left inferior frontal gyrus, and the occipito-temporo-parietal cortex (Martin and Chao, 2001; Patterson et al., 2007; Hayama and Rugg, 2009; Greenberg and Verfaellie, 2010; Binder and Desai, 2011).

Perhaps even more intriguingly, the thalamo-frontal network has been involved in future thinking. Clinical evidence in this respect is slim, yet Weiler et al. (2010c) documented two cases of patients with mediodorsal ischemic lesions, who showed deficits on a future thinking task. The task required subjects to provide a detailed account of future events in response to a verbal cue. Interestingly, one of the patients appeared free of recognition memory deficits. Weiler et al. (2010a,b) also provided fMRI evidence on the activation of the thalamus, the DLPFC, and the HC during future thinking.

In conclusion, there is strong anatomical evidence supporting a thalamic-PFC network centered in the MD. This network can exert its influence on cognition directly or through the interaction with other brain regions mediated by the PFC and the RTN. Evidence from clinical and neuroimaging studies highlights the importance of this network in episodic memory, particularly with respect to recall. The subunits of the MD show different connectivity patterns and also different activation patterns; however, clinical evidence in this regard is still very limited. Future studies need also to address more systematically whether the deficits are related to the encoding or retrieval phase of memory. It is crucial, in our view, to provide quantitative evidence on the lesions and the activations documented in the MD, and also to consider other components of the thalamo-PFC network (ILN, midline nuclei, RTN) when interpreting the data. Finally, the contribution of the thalamo-PFC system to episodic memory likely depends on task requirements. In light of the seemingly wide function of the thalamo-PFC network in cognition, we suggest that specifically the relevance to the behavioral goal is a variable to take into account in future experimental designs.

### The thalamo-retrosplenial network

In this review we refer by this name to the network that has been introduced by Aggleton and Brown (1999) and extensively characterized from anatomical and functional viewpoints over the last years (for reviews, see: Aggleton et al., 2000; Aggleton and Pearce, 2001; Aggleton, 2008, 2010, 2012; Vann et al., 2009a; Aggleton et al., 2010, 2011). We focused on the thalamus and the RSC for our definition to highlight that in the hypothesized information flow from the HC to the cortex these nodes seem to play a different role, compared to “pre-thalamic” regions. This stance is meant to highlight specializations within the functional unity of the network. In general, however, we use the term “thalamo-RSC” network to indicate the whole connectivity pattern including the HC, the fornix, the mammillary bodies, the MTT, the AT (including the laterodorsal nucleus), the thalamo-RSC connections, and the RSC (including the posterior cingulate cortex).

**Figure 3:**
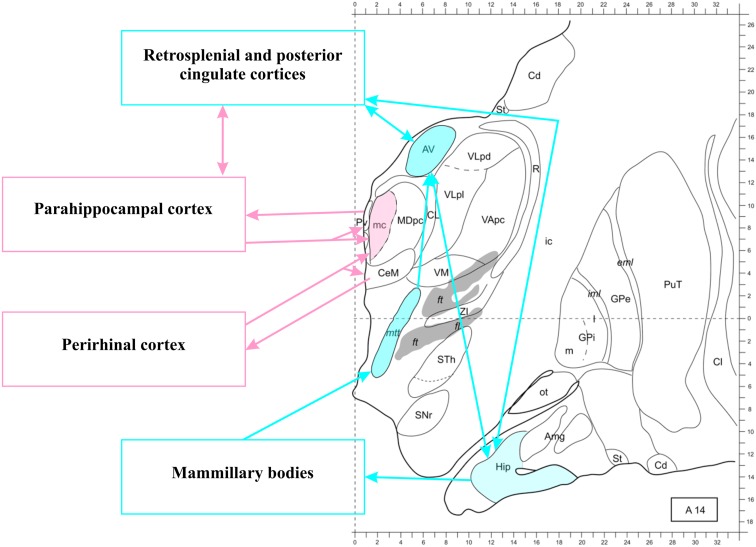
**The thalamo-retrosplenial cortical network**. Transversal section 14 mm anterior to the posterior commissura. The anterior thalamus, the hippocampus, and the retrosplenial/posterior cingulate cortices form a loop (see Aggleton, 2012, for further details). The magnocellular mediodorsal nucleus (MDmc) does not receive projection from the hippocampus, but from other structures of the medial temporal lobe (including the amygdala, as shown in Figure 2). The midline nuclei (not colored) receive and send back projections from all medial temporal lobe structures. Note that connections between medial temporal lobe structures are not shown, see Figure 1 for an illustration. Modified from Morel (2007). Abbreviations: Amg, amygdala; AV, anteroventral nucleus; Cd, caudate nucleus; CL, centrolateral nucleus; Cl, claustrum; eml, external medullary lamina; ft, fasciculus thalamicus; ic, internal capsula; iml, internal medullary lamina; GPe, globus pallidum, pars externa; GPi, globus pallidum, pars interna; Hip, hippocampus; MD, mediodorsal nucleus; MDpc, parvocellular MD; mtt, mammillothalamic tract; ot, olfactory tubercle; PuT, putamen; Pv, paraventricular nucleus; R, reticular thalamic nucleus; SNr, substantia nigra, pars reticulate; St, striatum; STh, subthalamic nucleus; VApc, ventral anterior nucleus, parvocellular portion; VLpd, ventrolateral nucleus, posterior dorsal subunit; VLpl, posterior lateral subunit; VM, ventromedial nucleus; ZI, zona incerta.

To briefly summarize the anatomy of the network, also schematized in Figure 3, hippocampal efferents run from the subiculum through the fornix to reach the mammillary bodies and the AT (Vann and Aggleton, 2004; Vann, 2010), even though some MTL fibers reach the anterior midline of the thalamus through the inferior thalamic peduncle (Aggleton, 2012). The mammillary bodies also project to the AT (Vann et al., 2007). The AT, in turn, sends direct projections to the HC through the fornix and also through the cingulum bundle (Aggleton et al., 2010), giving off collaterals in the cingulate cortex. An important intermediate station in the cingulate cortex is the RSC (Morris et al., 1999), which communicates reciprocally with the AT and the HC.

Although the network has been analyzed in greater detail in animal models, results from fMRI studies yielded consistent evidence for RSC activation during recognition memory, together with the lateral parietal cortex (Wagner et al., 2005; Pustina et al., 2012). These regions are thought to belong to the so-called “default mode network” (DMN), which also includes the HC and the ventromedial PFC, and is probably involved in associative processing (Raichle et al., 2001; Vincent et al., 2006; Bar, 2007; Bar et al., 2007; Mason et al., 2007). The existence of a default mode or resting state network was first discussed by Gusnard and Raichle (2001). The specific features of this network have been studied extensively since this time, the most consistent finding being that the DMN is more active during rest than during task performance. Bar et al. (2007) suggested that the DMN is also involved in processing contextual associations, and has thus been named “context network.” It has been shown that the DMN is recruited when subjects are using an associative strategy to encode items (Cavanna and Trimble, 2006; Peters et al., 2009). More in general, DMN regions have been reported to support stimulus-independent processing (McGuire et al., 1996; Christoff et al., 2004) and mind-wandering (Mason et al., 2007), although recent results more tightly link its activity to declarative memory (Shapira-Lichter et al., 2013). The thalamo-RSC network discussed in this review appears to be a subcomponent of the DMN network that is most convincingly related to episodic memory processing.

As far as recall is concerned, there is general agreement that integrity of all nodes and tracts of this pathway is critical (Park et al., 2007; Vann et al., 2007, 2009a,b; Vann and Albasser, 2009; Aggleton et al., 2010; Carlesimo et al., 2011). In spite of this consensus, the individual contributions of its components are still under investigation. The AT seem to exert its influence especially over the cingulate cortex and the RSC in particular. Garden et al. (2009) demonstrated decrease of synaptic plasticity in the RSC following AT lesions in rodents. Interestingly, no overt modulation of the electrophysiological response of single receptors occurred, suggesting indirect physiological effects. Evidence from studies targeting genetic expression shows that lesions to the AT result in under regulation of select genes’ expression in the RSC (reviewed by Vann and Albasser, 2009; Aggleton, 2010). The tight link between the AT and the RSC is supported by evidence that, across species, the degree of differentiation and the size of the AT correlates with the degree of differentiation in the RSC (reviewed by Jones, 2007). Since Aggleton and Brown (1999) proposal, the AT has been assumed to extend the hippocampal function. This tenet is certainly warranted and well-grounded on solid evidence. However, we will argue that the AT also shows a contribution to memory that is different from that operated by the HC, and perhaps more linked to the modulation they exert on the RSC.

#### Clinical evidence

The findings mentioned from animal models appear to extend to humans. It has been shown that RSC activity decreases after lesion in the AT (Fazio et al., 1992; Reed et al., 2003; Clarke et al., 1994). Amnesia following damage to the RSC is a well-known phenomenon (reviewed by Vann et al., 2009a; Aggleton, 2010). Damage to the AT also induces amnesia. Harding et al. (2000), in a post-mortem study, found that in alcoholics diagnosed with Wernicke–Korsakoff syndrome cell loss in the AT was the best predictor for amnesia. Disruption of the MTT and the fornix similarly causes recall deficits (Carlesimo et al., 2007; Cipolotti et al., 2008; Tsivilis et al., 2008; Rudebeck et al., 2009). Hence Aggleton and Brown (1999) and Aggleton et al. (2011) proposed that the thalamo-RSC network acts as a unitary recall system, and that damage to any node of the network will cause amnesia.

There is general agreement that lesion to the AT causes recall deficits that resemble the consequences of HC lesion, yet these deficits are surprisingly poorly characterized in neuropsychological literature. We systematically reviewed all stroke reports of memory deficits following *lesions to the AT with spared MD and sufficient neuropsychological assessment of memory*. Results are shown in Table 2 (see footnote 4, for the criteria used).

We were able to find only six studies that met our criteria, for a total of 16 patients. Data are so scarce because usually AT damage follows an infarct of the tuberothalamic artery; however, the same artery supplies the rostro-lateral part of the MD, especially the MDpc (Schmahmann, 2003). Damage to the MD is assumed to occur in particular following paramedian infarcts, but an analysis including 19 patients with thalamic stroke found no significant difference in the volume lost in the MD following paramedian and tuberothalamic stroke (Pergola et al., 2013c). This may help explain why memory deficits are similar between patients with different etiology (Pergola et al., 2012).

The cases available support the notion that lesion to the AT disproportionately impairs recall over recognition memory (Table 2, columns V and VI). Although the MD-PFC connections could have been involved because of damage to the anterior thalamic radiation, there was no evidence of direct damage to the MD in the cases reviewed.

Next, we asked whether these reports supported the assumption that damage to the AT disrupts the function of the HC. Based on the HC-AT connectivity, Van der Werf et al. (2003a) proposed that impairment after AT lesions derives from defective encoding. The RSC, instead, is mostly activated at retrieval (Wiggs et al., 1999; Huijbers et al., 2012, 2013), so we used neuropsychological clues of disrupted encoding or retrieval to inform our analysis.

Although the sample size is very small, 15 out of 16 cases showed evidence of retrieval deficits. Some cases showed encoding deficits only in the acute phase of the disease (i.e., the first week after lesion onset). Hence we found no published evidence of stable memory deficits that could be attributed to defective encoding following selective lesions to the AT. For 13 cases (Hanley et al., 1994; Ghika-Schmid and Bogousslavsky, 2000) the deficits were explicitly interpreted as retrieval-dependent. This conclusion, however, remains a working hypothesis in light of the confounds that also some of these studies present (Table 2, gray background) and because of the few cases available. We suggest that future clinical studies should employ tests that allow discrimination of encoding and retrieval deficits and/or use neuroimaging techniques to study the functional consequences of selective lesion to the AT.

#### Neuroimaging evidence

The phase of activation of the DMN regions, i.e., encoding or retrieval, is a currently debated controversy in the neuroimaging community. There is large consensus on the paramount role of the HC in episodic encoding; evidence on the involvement of the HC in retrieval is more conflicting, since only some of the numerous neuroimaging studies on the role of the HC in episodic memory did find significant activations during retrieval. When activation of the HC during retrieval was observed, an interference of incidental encoding processes during retrieval could not be ultimately ruled out (Stark and Okado, 2003; Huijbers et al., 2009).

Reas and Brewer (2013) performed a parametric analysis of BOLD activation during retrieval, using response times as a proxy for the duration of retrieval search. The authors found that activation of the HC was negatively correlated with response times. The same pattern applied to the medial PFC, posterior cingulate, and inferior parietal cortex. The same regions increased their reciprocal connectivity during successful *incidental* encoding. The authors interpreted this pattern as a deactivation induced by effortful retrieval, in the view of competition for cognitive resources between encoding and retrieval (Huijbers et al., 2009). Interestingly, however, the thalamus (anterior and medial, as can be judged based on Figure 4 by Reas and Brewer, 2013; no coordinates were reported) showed a positive correlation with response times, hence it was involved in effortful retrieval according to authors’ interpretation. This evidence suggests that in the thalamo-RSC network there is a parcellation of labor, with the thalamic node performing somewhat different operations compared to the HC and RSC.

Within the thalamus, different regions are activated in different phases of the memory process. The parcellation of the thalamic activation performed by Pergola et al. (2013b) revealed that the AT was selectively active during retrieval. The contrast used compared recognition cues characterized by post-scanning successful recall of the unique associations studied with cues characterized by no subsequent recall. Therefore the findings are in line with the role of the AT in recall. The activation appeared confined to the retrieval phase, and no voxels were activated at group level during encoding. The connectivity pattern of the activated voxels, assessed using established connectivity atlases, appeared consistent with the localization of the clusters in the AT. Finally and most importantly, activation in the putative region of the AT (defined on the basis of an anatomical atlas) correlated with individual recall scores during retrieval, and not during encoding. This evidence does not imply that the AT are inactive during encoding: it is possible that the AT were equally active during successful and unsuccessful encoding, hence preventing detection of significant clusters. However, the finding that inter-individual variability in the activation of the AT during retrieval correlates with recall performance suggests that the AT play an autonomous role during this phase of memory processing.

As we also argued for the thalamo-PFC network in Section “The Thalamo-Prefrontal Network,” the thalamo-RSC network has been implicated in wider cognitive functions than recall. It is debated whether the involvement of the DMN is exclusive of memory, or extends to endogenous processing more in general. Tasks without overt demand for memory processing appear to recruit the DMN, including for instance theory of mind (Buckner and Carroll, 2007) and self-referential processing (Johnson et al., 2002). The neural network that supports semantic knowledge related to the self includes the anterior and posterior cingulate cortices as well as the RSC (Gobbini et al., 2004; Donix et al., 2010). However, the same areas are activated even to a greater extent, together with the anteromedial PFC, by episodic autobiographical memory (Levine, 2004; Levine et al., 2004). A recent meta-analysis on neural correlates of autobiographical memory highlighted the common participation of the thalamus, the RSC, the anterior cingulate, and the medial PFC in both episodic and semantic aspects of self-referenced memory (Martinelli et al., 2012). By comparing directly mnemonic and non-mnemonic tasks, Shapira-Lichter et al. (2013) could show that the RSC was more activated during mnemonic processing, both episodic and semantic.

These pieces of evidence suggest that the thalamo-RSC network could constitute a possible *trait d’union* between episodic and semantic memory; on the other hand, they also point to differences in the contribution of the individual nodes of the network to memory. We suggest that the interaction between the AT and the RSC in particular deserves more investigation in humans, and we put forward working hypotheses on the functional specialization of these regions in the next section.

#### Models on the functional architecture of the thalamo-RSC network

Functional dissociations between the nodes of the thalamo-RSC network have been examined by Huijbers et al. (2012). Authors highlighted that the posterior cingulate and retrosplenial regions include a number of different areas that also show different connectivity patterns, and for this reason several models fail to account exhaustively for RSC activation in fMRI studies. Given that also the AT present heterogeneous connectivity patterns with the hippocampal subfields (reviewed by Aggleton, 2012), it is possible that the thalamo-RSC network consists of multiple pathways partially segregated with respect to their connectivity and function.

In general, the thalamo-RSC network, as part of the DMN, likely subserves associative memory and particularly recall, as documented by a large body of evidence. Work with animal models suggested that the commonalities between impairments observed following HC and AT damage may depend on the particular task used to assess memory (Sziklas and Petrides, 1999, 2004); it is also possible that the RSC constitutes a non-fornical pathway for bilateral communication between the HC and the AT (Henry et al., 2004; Dumont et al., 2010). Evidence from animal studies therefore presents commonalities and differences with respect to the HC, AT, and RSC contribution to memory. Evidence from clinical and neuroimaging studies suggests that the phase of activation – i.e., retrieval – of the AT and RSC is different from what is typically found for the HC, i.e., encoding.

It has thus been suggested that the AT and the RSC are involved in activating and maintaining stored perceptual representations during retrieval (von Zerssen et al., 2001). This proposal would explain why cued recall is required to elicit this activity: recognition by itself does not require activation and maintenance of perceptual representations. This hypothesis assumes a role of the AT during retrieval that is consistent with the evidence reviewed. Vilberg and Rugg (2012) studied the different activation patterns between transient and sustained activation as a function of the duration of maintenance of the representations (analysis focused on the retrieval phase). They did not find activations in the thalamus (although this may partly depend on scanning parameters), but they found that the HC and the RSC were only transiently activated during retrieval. Their findings therefore do not lend direct support to the idea that the HC-RSC network contributes to representation maintenance.

Another intriguing possibility is provided by the Multiple memory Trace Theory (Moscovitch et al., 2005), which predicts that memory retrieval entails the cumulative generation of multiple memory traces that somewhat differ from the original memory trace. In particular, new memory traces are more schematic and more dependent on the RSC (Hirshhorn et al., 2012), while they become progressively independent from the HC. This theoretical framework requires the existence of brain regions intermediate between the HC and the neocortex that transform the memory traces and establish the information originally encoded by the HC into neocortical areas. Also on the basis of neurophysiological findings reviewed below, we propose that the features of the AT (in particular, the regulation of RSC plasticity) match the requirements to subserve this function. In this view, the AT-RSC connections would underlie the generation of multiple memory traces, a role that includes retrieval processes (of the original memory trace) and re-encoding (generation of novel memory traces); on the cognitive side, this information flow could be a relevant path for the conversion of episodic into semantic memory traces. Testing this working hypothesis will require a deeper understanding of the temporal dynamics of network activations, in line with the approach followed by Vilberg and Rugg (2012). Rather than focusing on the magnitude of activations in the nodes of the network, it is necessary to collect more information on the duration of the activations – an approach that would match the focus on long-term potentiation (LTP) found in neurophysiology.

## Consequences of Disruption of the Thalamo-PFC and Thalamo-RSC Networks in Psychiatric Disorders

Thalamic neuropathology, especially of ischemic and degenerative etiology, has been thoroughly studied in connection with memory deficits (reviewed by Kopelman, 2002; Schmahmann, 2003; Carrera and Bogousslavsky, 2006; Carlesimo et al., 2011). More recently, evidence is accumulating that also psychiatric conditions might include thalamo-cortical dysfunction as a salient feature of their neuropathological picture.

Patients with schizophrenia, in particular, show structural and functional abnormalities in the HC, PFC, and the thalamus. The cognitive profile that accompanies the structural and functional peculiarities of schizophrenia is characterized, among other symptoms, by episodic memory deficits that constitute one of the most impaired aspects of the cognitive profile (Saykin et al., 1991; Mitropoulou et al., 2002; D’Argembeau et al., 2008; Minzenberg et al., 2009). The episodic memory impairment is more evident on recall than on recognition (Pelletier et al., 2005; Thoma et al., 2006; Libby et al., 2013). The weak dependence of the deficits on the duration of the study-test delay led to the hypothesis that the impairments relate to encoding rather than retrieval (Aleman et al., 1999; Gold et al., 2000; Dickinson et al., 2007).

The deficits displayed by patients with schizophrenia are thus suggestive of a dysfunctional thalamo-PFC network (Kuperberg, 2008; Mitchell and Johnson, 2009; Blumenfeld et al., 2011; Libby et al., 2013). Beside the amount of data involving the PFC in the pathology (reviewed by Bertolino and Blasi, 2009), post-mortem studies found specific cell loss in the MD of patients with schizophrenia (Young et al., 2000; Byne et al., 2002), and especially of its parvocellular portion (Popken et al., 2000; reviewed by Byne et al., 2009). Other reports highlighted altered metabolism in the MD and ILN of patients with schizophrenia (Hazlett et al., 2004). Drugs targeting the D2 dopaminergic receptor improve the symptomatology, and the MD presents a high density of D2 receptors (Rieck et al., 2004; Vogt et al., 2008). This feature characterizes primates compared to rodents (Garcia-Cabezas et al., 2009). In healthy subjects, low density of D2 receptors in the thalamus has been related to high creativity in a positron emission tomography study (de Manzano et al., 2010). Dysfunction and decreased connectivity of the MDmc and the orbitofrontal PFC to which it projects have been related to the psychotic symptoms of schizophrenia (Popken et al., 2000; Kubota et al., 2013). The involvement of the MDpc, on the other hand, has been claimed to relate to the cognitive impairments shown by patients with schizophrenia (reviewed by Alelú-Paz and Giménez-Amaya, 2008; Pakkenberg et al., 2009).

Interestingly, the critical alterations in the brain of patients with schizophrenia that affect the networks described in this review, and the thalamo-PFC in particular, are not related to acute damage in a single node of the network. Schizophrenia has been characterized as a neurodevelopmental disorder. In this framework, symptoms of schizophrenia can be thought of as a model of progressive thalamo-cortical dysfunction, with a strong genetic component (Bertolino and Blasi, 2009; Blasi et al., 2013). Accordingly, the neuroimaging literature is rich of evidence on dysfunctional activation and connectivity of the thalamo-PFC network in patients with schizophrenia and also in healthy controls with genetic risk for schizophrenia (Hariri et al., 2003; Bertolino et al., 2008; Di Giorgio et al., 2012; Anticevic et al., 2013). Functional connectivity studies have established altered coupling between the HC and the lateral PFC as a correlate of genetic risk for schizophrenia (Bertolino et al., 2006; Tunbridge et al., 2013).

Altered coupling of the HC and the DLPFC is a notable finding, because the HC is more strongly connected to the ventromedial and orbitofrontal PFC, than to the DLPFC. Based on the thalamo-cortical networks outlined in this review, it is plausible that a transthalamic route involving the MDpc, the RTN, and the MDmc/midline nuclei mediates the interaction between the two brain regions. Cholvin et al. (2013) found evidence of this functional circuitry in rodents; Klingner et al. (2013) obtained results consistent with this suggestion using resting state fMRI. The MDpc-DLPFC connectivity could modulate activity in the RTN, which is able to regulate the midline thalamic nuclei directly projecting to the HC. Matching this hypothesis, Dauvermann et al. (2013) showed decreased MD-PFC connectivity during a verbal fluency task in healthy participants with high genetic risk for schizophrenia, as assessed through non-linear Dynamic Causal Modeling. The effect was greatest for patients also showing symptoms of psychosis.

In summary, the study of the pathophysiology of schizophrenia offers the opportunity of further insight into the physiological and molecular properties of the thalamo-PFC and the thalamo-RSC networks. The next section will go more in detail in the putative mechanisms that might underlie their role in episodic memory.

## Possible Mechanisms of the Thalamo-Cortical Contribution to Memory and Related Pathologies

The thalamus is considered to serve as an interface between subcortical structures and the cortex and basal ganglia. It has been described as a “searchlight” (Crick, 1984; Smythies, 1997), an “enhancer” (LaBerge, 1997), and a “focuser” (Van der Werf et al., 2003a). Research has partially discounted the unitary views in recent years, placing emphasis on the diversity of the thalamic nuclei (Sherman and Guillery, 2002).

In this respect it is noteworthy that some thalamic nuclei receive their driving inputs from non-cortical regions (first-order nuclei; e.g., the AT), while other nuclei, such as the MD and the pulvinar, receive their driving input from the cortex, particularly from the V cortical layer. Since these driver inputs do not synapse in the RTN, but they do synapse on subcortical effector nuclei, it has been proposed that these cortico-thalamic connections bear information on planned actions, that is then relayed by the thalamus to other cortical regions (Guillery and Sherman, 2011; Sherman and Guillery, 2011). This information route is called “transthalamic” and is hypothesized to supply upstream cortical regions with information about the signals they are about to receive from downstream cortical regions, particularly with respect to action execution (Byne et al., 2009). Given the size and conductance of cortico-thalamic and thalamo-cortical fibers it is even possible that the transthalamic route is faster than direct cortico-cortical communication (Salami et al., 2003). The next paragraphs will briefly sketch possible mechanisms of the thalamo-cortical contribution to memory, with mention of the most recent evidence on the topic.

### Pacemaker

The connectivity pattern of the MD enables it to mediate transthalamic communication between temporal and prefrontal regions, as well as between lateral prefrontal subregions. The role of thalamic nuclei in entraining cortical oscillations is well-recognized (Steriade, 2006; Sherman, 2007), and more recent evidence supports the involvement of the MD in particular in regulating PFC oscillations during memory processes. By using a procedure to reversibly disconnect the MD and the PFC in mice, Parnaudeau et al. (2013) demonstrated memory deficits in a delayed non-matching to sample task at long delays (see Aggleton and Brown, 1999, for a discussion about the use of this task to assess recognition memory in rodents). Performance was impaired in trained animals, suggesting that the deficit was not limited to the encoding phase. The critical range of frequencies for the MD-PFC connectivity was the beta and gamma range.

The dynamics of the MD-prefrontal interplay in humans have been recently reported by Staudigl et al. (2012), who studied a patient with epilepsy by means of intracranial recordings. The authors demonstrated a link between thalamic activity in the putative MD territory during retrieval and scalp frontal beta-frequency modulation in the time window 300–500 ms post-stimulus onset. This time window is commonly associated to a frontal old/new effect in the ERP literature (reviewed by Paller et al., 2007; Rugg and Curran, 2007). Strikingly, Staudigl et al. (2012) could show that the direction of the signal was thalamo-cortical. Additionally, the authors reported cross-frequency coupling relating the power in the beta range with power in the gamma frequency-range (Staudigl et al., 2012). Unfortunately, the study did not report activity during encoding and concluded that the MD is more involved in retrieval compared to the AT, a finding to our knowledge not supported by any evidence based on studies with humans (as discussed, see The Thalamo-Retrosplenial Network). Notwithstanding the difficulties of locating electrodes in the thalamus of the epileptic patient, the AT are first-order nuclei projecting to deep brain regions, so several synapses could be needed before the signal reaches the outer cortex, which is the main source of scalp potentials. Fitzgerald et al. (2013) extended these findings by showing a more complex pattern of functional connectivity between the thalamus and the PFC. The interactions included phase-amplitude and amplitude-amplitude coupling and were established based on data from three patients receiving deep brain stimulation.

This evidence suggests that the contribution of the thalamo-PFC network to episodic memory can be mediated by modulation of cortical oscillations induced by thalamic activity. Disruption of thalamo-PFC connectivity also characterizes schizophrenia: in the study by Anticevic et al. (2013), the most prominent locus of thalamic dysconnectivity was centered on the MD.

### Plasticity device

Aggleton et al. (2011) proposed that the AT may be part of a triangular circuitry including also the HC and the cingulate and prefrontal cortices. Triangular connections enable the setup of coincidence detection systems (Jones, 2007) based on LTP. In rodents, LTP can be induced in the cingulate and PFC by co-activation of convergent hippocampal and thalamic afferents (Gigg et al., 1992, 1994). Further evidence from animal studies suggests that the AT modulates plasticity in the RSC (Garden et al., 2009). As previously mentioned, it can be hypothesized that the cingulate (especially RSC) and medial prefrontal LTP could foster transfer of memory traces from the HC to the neocortex during retrieval and concurrent re-encoding (Moscovitch et al., 2005; Hirshhorn et al., 2012). A proof of concept for this working hypothesis comes from a study with rats in which modulation of the MD-PFC connectivity was shown to affect prefrontal LTP, resulting in strengthening or decreasing thalamo-cortical connectivity (Bueno-Junior et al., 2012).

Long-term potentiation could therefore be one physiological mechanism underlying the role of thalamo-cortical networks, and especially the thalamo-RSC network, in episodic memory. It is established that LTP is a critical feature of memory formation, and evidence is accumulating in favor of a “plasticity account” for diseases such as schizophrenia (reviewed by Weinberger and Harrison, 2011).

A critical feature of a plasticity regulator is selectivity: enhancing or decreasing signal in general would not be sufficient to afford a selective increase of specific patterns of neural activity. The thalamus, as a complex, is particularly suitable for such a role. The thalamus sends selective as well as widespread projections to the cortex, ending in specific cortical layers (Byne et al., 2009). The convergence of specific (e.g., MD to PFC) and aspecific connections (e.g., ILN to PFC) would provide the basic circuitry necessary for a coincidence detection system (see the core/matrix hypothesis, Jones, 2007) that would be able to modulate plasticity in specific cortical populations. The potential importance of such processes for memory has been shown by Logothetis et al. (2012), who found that increased activation in the HC was accompanied by decreased thalamic activation. The authors proposed that decreased thalamic activity may serve to shield the cortex from interference during memory consolidation. This proposal is particularly intriguing in light of the findings that in the acute phase of ischemia involving the AT patients are especially sensitive to interference (see Table 2).

In summary, the effect of the thalamo-cortical interaction could operate at a greater time scale than previously thought, determining long-term changes in connectivity, a suitable mechanism for memory formation and transformation.

## Summary

In this article we summarized evidence that beside the medial temporal lobe, two other brain networks are involved in memory processing. These structures are not exclusively bound to memory processes, but also contribute to executive functions and future thinking. The evidence reviewed shows that memory processing at different stages draws on different and also shared resources depending on the required processes.

The thalamo-PFC network has often been mentioned as fundamental for declarative, and especially episodic, memory. Until now, however, it is debated whether it rather plays only an ancillary role with respect to the MTL. We argued that by using tests that specifically tap recall it is possible to reveal the contribution of the thalamo-PFC network to episodic memory. Differently from previous views, we argued that the MD is critical for recall. The field has not sufficiently investigated whether subunits of the MD are functionally segregated, and this remains a task for future experimental scrutiny. Our working hypothesis is that this network is especially involved in goal-directed, as opposed to incidental, memory acquisition. This hypothesis can be tested by comparing incidental against goal-directed encoding, and also by comparing results obtained through tasks that induce different cognitive orientations. Regulating medial temporal and prefrontal oscillations and connectivity are possible mechanisms by which the network could act; these mechanisms have been shown to be dysfunctional in schizophrenia, which in many aspects shows a neuropathophysiology consistent with disruption of the thalamo-PFC network.

The importance of the thalamo-RSC network in recall has met wide consensus. We reviewed evidence that the understanding of the functional unity of this network that dominated the recent literature is changing, following recent discoveries on functional specializations of its nodes. The recruitment of different nodes of the network in different memory stages (encoding and retrieval) is a consistent finding. In particular, clinical and neuroimaging evidence indicates an involvement of the AT and the RSC in the retrieval phase of memory processing. The functional parcellation of the network is particularly interesting in light of models of memory, such as the Multiple Memory Trace theory, that posit the existence of regions governing the information flow from the HC to the neocortex. The thalamo-RSC network appears to be especially fit for this function, possibly mediated by regulation of neocortical plasticity. This hypothesis can be tested by focusing on the duration of activations and on the effects of repeated exposure to stimuli, whose representation passes from episodic- to semantic-like.

We suggest that advancing the field will require a more extensive use of quantitative procedures in the assessment of thalamic lesions and activations, and we share the impression of Metzger et al. (2013) that improving the spatial resolution of imaging methods will greatly enhance our understanding of the function of non-MTL regions in memory. Moreover, we suggest that focusing research on different aspects of recall and their neural determinants rather than on recall/recognition dichotomies will be an effective way to move forward. We are still bound to the statement by James reported at the beginning of this review about empirical design of memory studies: recall seems to be the chief task to study episodic memory.

## Conflict of Interest Statement

The authors declare that the research was conducted in the absence of any commercial or financial relationships that could be construed as a potential conflict of interest.

## Footnotes

^1^For the sake of simplicity the term “hippocampus,” abbreviated with HC, refers throughout the article to the hippocampal formation, including the dentate gyrus, CA fields, subiculum, presubiculum, and parasubiculum. Aggleton (2012) goes more in detail with respect to the connectivity of HC subregions and should be consulted for further reference.

^2^Throughout the review we will endorse the viewpoint expressed by Jones (2007) that other partitions of the MD (densocellular, paralamellar, or multiformis) rather belong to the centrolateral nucleus, which is considered part of the ILN.

^3^As specified in the previous footnote, this group of thalamic nuclei includes the densocellular and paralamellar (or multiformis) partition of the MD in the current review.

^4^Articles were considered based on previous reviews and on the PubMed search: (thalam*[title/abstract]) AND (stroke[title/abstract] OR infarct[title/abstract] OR ischemia[title/abstract] OR ischaemia[title/abstract] OR ischemic[title/abstract]) AND (memory[title/abstract] OR learning[title/abstract] OR recollection[title/abstract] OR recognition[title/abstract] OR familiarity[title/abstract] OR amnesia[title/abstract] OR amnesic[title/abstract]) AND english[language].
